# Defenders and Challengers of Endothelial Barrier Function

**DOI:** 10.3389/fimmu.2017.01847

**Published:** 2017-12-18

**Authors:** Nader Rahimi

**Affiliations:** ^1^Department of Pathology, Boston University School of Medicine, Boston, MA, United States

**Keywords:** cell adhesion molecules, vascular permeability, endothelial dysfunction, vascular endothelial growth factor A, adherens junctions, gap junctions

## Abstract

Regulated vascular permeability is an essential feature of normal physiology and its dysfunction is associated with major human diseases ranging from cancer to inflammation and ischemic heart diseases. Integrity of endothelial cells also play a prominent role in the outcome of surgical procedures and organ transplant. Endothelial barrier function and integrity are regulated by a plethora of highly specialized transmembrane receptors, including claudin family proteins, occludin, junctional adhesion molecules (JAMs), vascular endothelial (VE)-cadherin, and the newly identified immunoglobulin (Ig) and proline-rich receptor-1 (IGPR-1) through various distinct mechanisms and signaling. On the other hand, vascular endothelial growth factor (VEGF) and its tyrosine kinase receptor, VEGF receptor-2, play a central role in the destabilization of endothelial barrier function. While claudins and occludin regulate cell–cell junction *via* recruitment of zonula occludens (ZO), cadherins *via* catenin proteins, and JAMs *via* ZO and afadin, IGPR-1 recruits bullous pemphigoid antigen 1 [also called dystonin (DST) and SH3 protein interacting with Nck90/WISH (SH3 protein interacting with Nck)]. Endothelial barrier function is moderated by the function of transmembrane receptors and signaling events that act to defend or destabilize it. Here, I highlight recent advances that have provided new insights into endothelial barrier function and mechanisms involved. Further investigation of these mechanisms could lead to the discovery of novel therapeutic targets for human diseases associated with endothelial dysfunction.

## Introduction

To live and reproduce, all vertebrate animals are evolved to have a circulatory system (i.e., heart, veins, and arteries) that safeguards an uninterrupted supply of blood and oxygen to all tissues, followed by the return of the deoxygenated blood to the lungs for re-oxygenation. In addition to its emissary function, the vascular system also plays an indispensable role in hemostasis, immune surveillance, angiogenesis, and vascular permeability (1). Although they differ in function and morphologies, endothelial cells are the main constituents of blood vessels. In some organs such as the brain, endothelial cells form a strong and highly selective blood–brain barrier, but in other organs such as the kidney and pancreas, endothelial cells display selective permeability by forming highly specialized holes on the plasma membrane called fenestrae, which allows rapid exchange of solute and molecules such as hormones.

To maintain the structural and functional integrity that retains the highly dynamic barrier function of blood vessels, which permits continuous leakage of solutes and small molecules but limits extravasation of larger molecules and cells, metazoan cells are evolved to form highly specialized cell–cell junctions such as desmosomes, adherent junctions, and gap junctions. Not only do these junctions glue cells together, they also generate intracellular signaling and permit junctional remodeling in response to various external and internal cues (2). Curiously, certain viruses employ cell adhesion receptors for their entry into human cells. For example, hepatitis C virus (HCV) uses occludin and claudins to enter liver cells. Coxsackievirus and adenovirus use junctional adhesion molecule (JAM)/coxsackie and adenovirus receptor (CAR) and reoviruses uses JAM-A for their entry into cells [for review see Ref. (3)]. In many human diseases, such as cancer, diabetes, age-related macular degeneration, and chronic inflammatory conditions, this core barrier function of endothelial cells breaks down, leading to the leakage of larger molecules and blood with serious life-threatening consequences. Blood vessel leakiness also is associated with tumor-induced angiogenesis and represents a significant challenge for an effective delivery of anti-cancer drugs to the site of tumors as tumor-associated blood vessels are structurally fragile and hyperpermeable (4).

**Figure 1:**
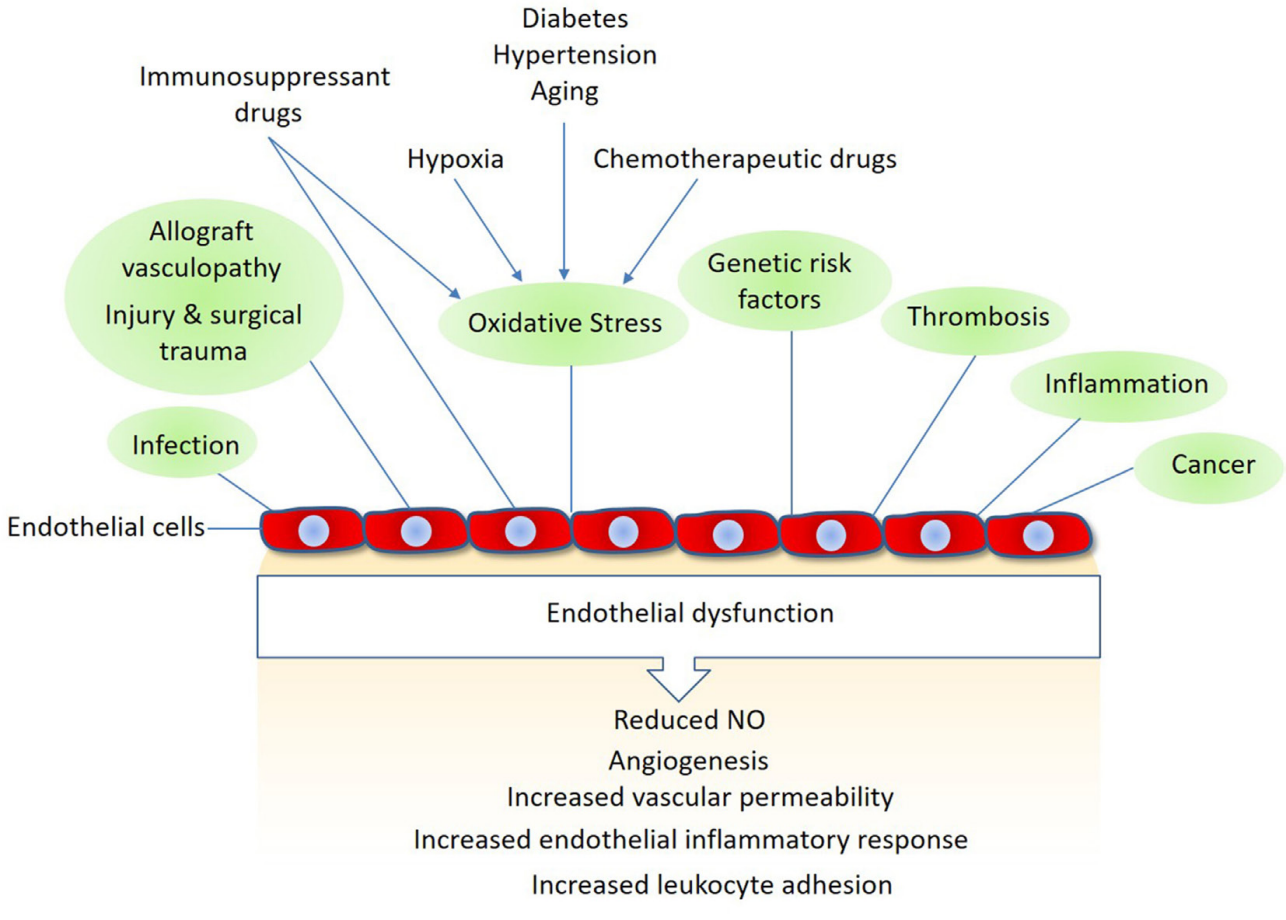
Mechanisms linked to endothelial dysfunction: several key mechanisms that promote endothelial dysfunction and vascular damage are shown. Also, shown are the major endothelial responses triggered by these factors Nitric oxide (NO).

In addition to their pivotal roles in angiogenesis and inflammation, endothelial cells also play important functions in various other conditions such as surgical trauma, ischemia–reperfusion, alloimmune responses, and chemotherapy and immunosuppressant treatments (5, 6). Activated endothelial cells often upregulate expression of various growth factors, cytokines and chemokines that stimulate endothelial cell proliferation, permeability, and migration (7, 8). Furthermore, they upregulate thrombogenic molecules and specific adhesion molecules that promote thrombosis and immune cell activation (Figure 1). Endothelial cells also respond to immunosuppressant and chemotherapeutic drugs. Although the cardiotoxic effects of conventional chemotherapeutic agents are well-documented, the targeted therapeutic drugs such as the antiangiogenic are also associated with endothelial dysfunction, such as hypertension, thromboembolism, myocardial infarction, and proteinuria (9, 10). In organ transplantation, the host immune system is brought into direct contact with the endothelial cell lining of graft vessels, where the graft endothelial cells play a major role in allograft vasculopathy (i.e., allograft rejection) and in the overall long-term survival after any organ transplantation (11, 12).

## Regulation of Endothelial Cell–Cell Junctions by Cell Adhesion Molecules (CAMs)

**Figure 2:**
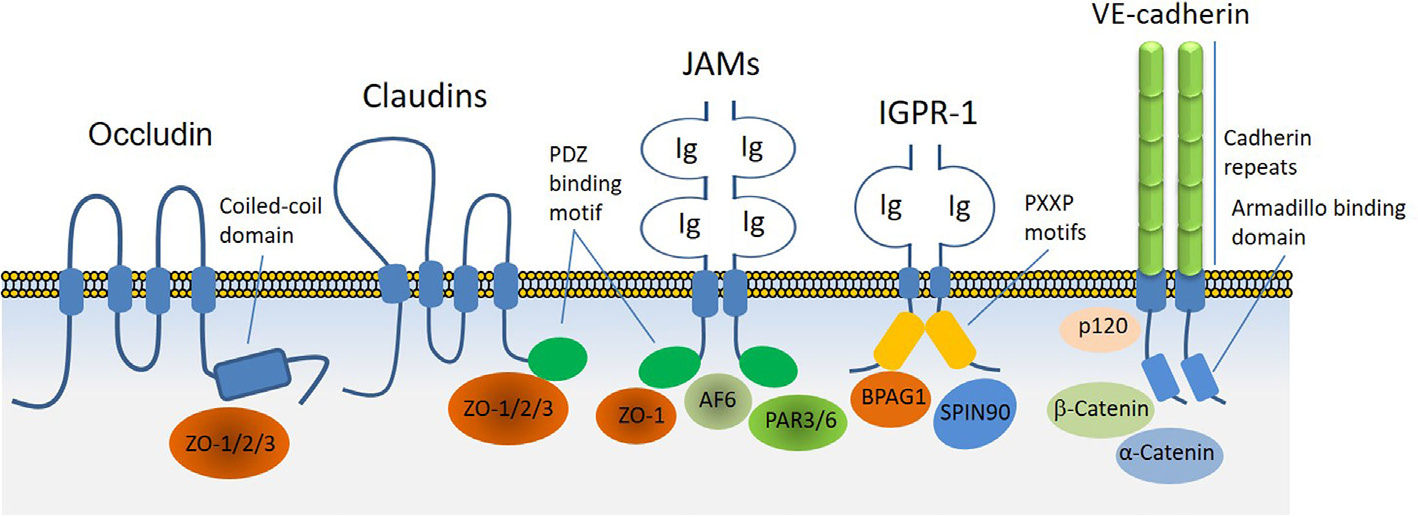
Transmembrane receptors involved in the endothelial cell–cell junction and their key cytoplasmic-binding partners. Claudin family proteins, occludin, junctional adhesion molecules (JAMs), vascular endothelial (VE)-cadherin an immunoglobulin (Ig) and proline-rich receptor-1 (IGPR-1) are major receptors in endothelial cells that regulate endothelial cell–cell junctions and barrier. The core mechanism associated with the function of these receptors involves with their ability to recruit specific signaling proteins that signal to strength the cell–cell junctions. Zonula occludens (ZO 1–3), catenin proteins (α-catenin, β-catenin, and γ-catenin), polarity protein-3 and 6 (PAR3/6), afadin (AF6), bullous pemphigoid antigen 1 [BPAG1 also called dystonin and SH3 protein interacting with Nck90 (SPIN90)/WISH (SH3 protein interacting with Nck)] are key substrates involved with these receptors.

Adherens junctions, gap junctions, and desmosomes are principal cell–cell junctions that provide structural integrity and create highly polarized barriers with selective paracellular permeability to solutes, macromolecules, and other cells, which is an essential element of homeostatic maintenance in endothelial and epithelial cells. Tight junctions, in particular, control monolayer permeability and play a significant role in endothelial cells that maintain rigorous barriers, whereas adherens junctions partake in multiple roles, such as establishment and maintenance of cell–cell adhesion, actin cytoskeleton remodeling, signal transduction, and transcriptional regulation. However, unlike epithelial cells, adherens and tight junctions in endothelial cells are highly interconnected. In addition to their cardinal role in the regulation of homeostatic maintenance and barrier function, proteins involved in the regulation of cell–cell junctions play major role in cellular differentiation, proliferation, migration, signal transduction, and gene expression (13, 14). Altered cell–cell junctions are also associated with the pathogenesis of various diseases, including cancers, diabetic retinopathy, and inflammation (15, 16). A plethora of cell surface receptors including claudins family proteins, occludin, JAMs, vascular endothelial (VE)-cadherin, and the recently identified immunoglobulin (Ig) and proline-rich receptor-1 (IGPR-1) are involved in cell–cell junction signaling through various means and mechanisms. While occludin through its cytoplasmic coiled-coil (CC) domain interacts ZO proteins, claudins family proteins and JAMs through their PDZ-binding motif interact with PDZ-containing proteins such as ZO. JAMs also interact with PAR3, PAR6 and AF6, which are also PDZ-containing proteins. On the other hand, VE-cadherin through its armadillo-binding domain recruits p120, catenin proteins, whereas IGPR-1 through its proline-rich motif interacts with (BPAG1 or BP230), also called dystonin (DST) and SH3 protein interacting with Nck90 (SPIN90)/WISH (SH3 protein interacting with Nck), also called NCK-interacting protein with SH3 domain (NCKIPSD) (Figure 2). Regardless of their mechanisms of recruitment of cytoplasmic-binding partners, it is clear that these receptor-interacting proteins transduce signals that are required for cell–cell junction assembly, cell morphology, and barrier function. In a way, these transmembrane receptors along with their intracellular-binding partners are the defenders of endothelial integrity and barrier function.

### Claudins

Claudin family proteins are four-transmembrane type proteins and there are at least 24 claudins present in human genome (17, 18), which represents the largest family of tight junction proteins. By forming homophilic- and heterophilic-*trans*/*cis* dimerization, claudins determine the barrier properties and cell–cell interactions (19, 20). Claudins, with the exception of claudin-12, contain a PDZ-binding domain at C-terminal tail that allows them to interact with PDZ-containing scaffold proteins such as zonula occludens (ZO) (19). It appears that unlike cadherins, claudins mediate cell–cell adhesion through a calcium-independent manner (20). Claudins are expressed in both endothelial and epithelial cells, though with some degree of cell type specificity. Claudin-3, claudin-5, and claudin-12 are predominantly expressed in brain endothelial cells (21, 22), whereas renal endothelial cells express claudin-5 and claudin-15 (21, 23). Some claudins such as claudin-2 and claudin-16 specifically control paracellular ionic selectivity by forming ion channels (24, 25), while others such as claudin-8 is proposed to control paracellular Na+ permeability (26). In addition to their canonical function, some claudins also interact with other proteins. For example, claudin-1 acts as a receptor for HCV (27) and for dengue virus (28).

### Occludin

**Figure 3:**
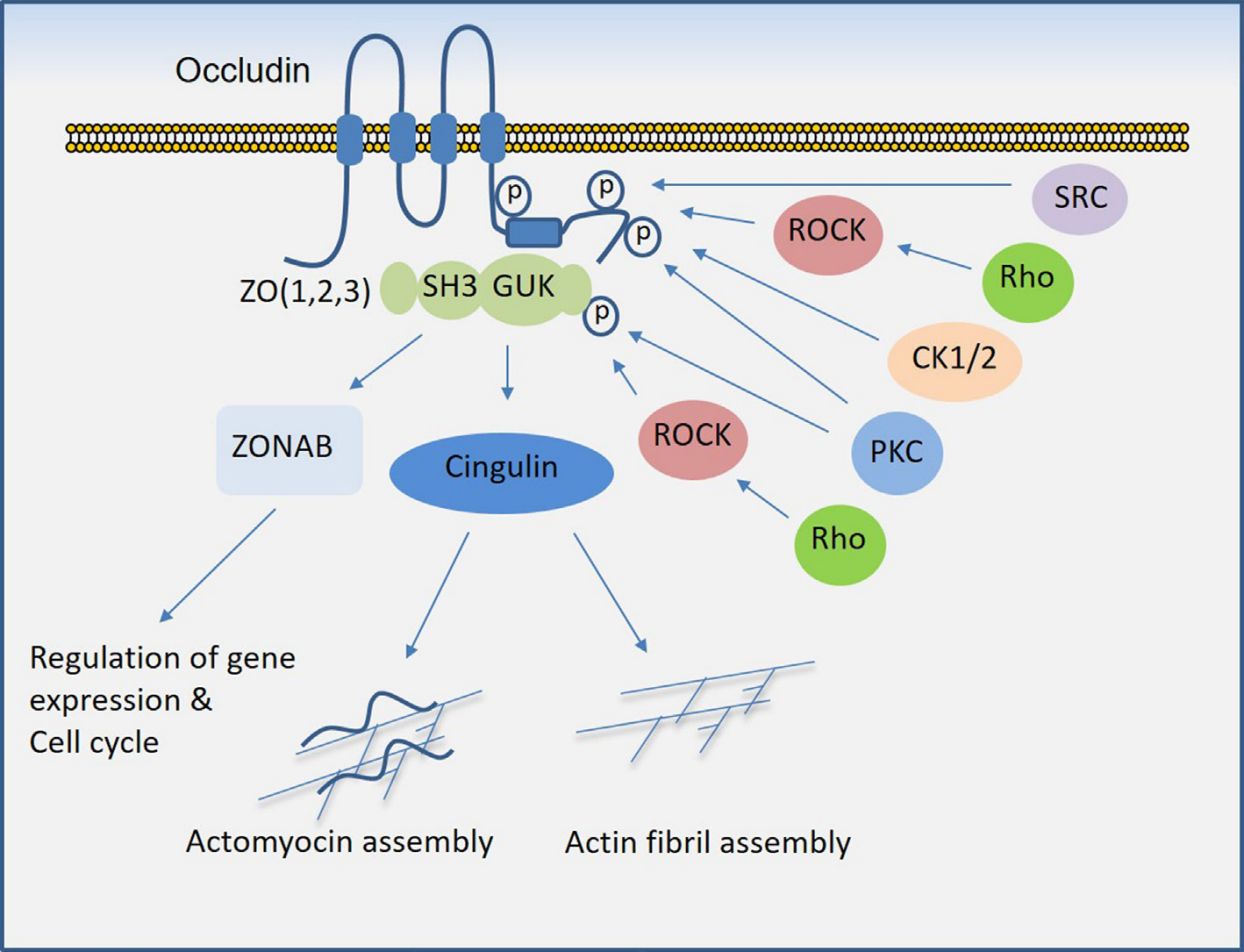
Regulation of occludin mediated cell–cell junction assembly and organization. Occludin *via* its coiled-coil domain recognizes guanylate kinase (GUK) domain, PDZ, and SH3 domain (43), that further recruits Cingulin and ZONAB that participate in the formation and regulation of the tight junction and paracellular permeability barrier. C-terminal of occludin is subject to phosphorylation at serine/threonine and tyrosine residues by multiple serine/threonine kinases and tyrosine kinases, which in part regulate occludin binding with ZO proteins and its tight junctional function (see the text).

Similar to claudins, occludin is a four-transmembrane protein and one of the key components of tight junctions that plays a critical role in the regulation of trans-epithelial/endothelial electrical resistance (29, 30) and actin assembly (31). While the N-terminal extracellular domain is involved in the adhesive function of occludin, its C-terminal is subject to phosphorylation at several tyrosine and serine/threonine residues through multiple kinases and is also involved in the recruitment of SH3 and PDZ-containing zonula occluden (ZO) proteins, which anchor occludin to the actin fibril assembly (Figure 3). In endothelial cells, it regulate tight junction barriers in response to IFNγ and vascular endothelial growth factor (VEGF) (32–34). The barrier function of occludin is regulated by the phosphorylation of key residues at the cytoplasmic domain (19, 35). For example, phosphorylation of Ser490 was proposed to promote ubiquitination of occludin, which promotes its downregulation (36), whereas phosphorylation of occludin at different sites is associated with its barrier function (37–39). The key kinases involved in the phosphorylation of occludin are shown (Figure 3). The interaction of occludin with tight junction proteins such as ZO family proteins is also affected by phosphorylation at its CC domain (40, 41). Overall, phosphorylation of the C-terminal of occludin at serine/threonine and tyrosine sites by various kinases (Figure 3) and dimerization (not shown) of occludin appear to be key mechanisms that govern occludin function (42).

### Junctional Adhesion Molecules

Junctional adhesion molecules are distinct and important cell surface proteins that are involved in the regulation of cell–cell adhesion and barrier. JAMs belong to the Ig superfamily proteins and contain two extracellular Ig-like domains, a single transmembrane domain and a C-terminal cytoplasmic domain (44). The cytoplasmic domain of JAMs contains a PDZ domain, which recruits PDZ-binding proteins such ZO and afadin that connects JAM proteins to actin assembly and regulation of epithelial and endothelial barrier function (44–46). JAM-A regulates the barrier function of tight junctions in both endothelial and epithelial cells (47) and is involved in the migration of endothelial cells (48). JAM-C was proposed to be involved in tumor angiogenesis (49). Furthermore, other JAMs such as CAR and endothelial cell-selective adhesion molecule are also expressed in endothelial cells and are involved in the regulation of permeability, angiogenesis, and cell migration (50, 51).

## Mechanisms of Destabilization of Endothelial Barrier Function

The control of the endothelial barrier function is largely mediated by cell-to-cell junctions, which include adherens and tight junctions. CAMs are the key mediators of endothelial barrier function. CAMs mediate cell–cell and cell–matrix adhesion and transmit signals across the plasma membrane to process information from the extracellular environment involved in tissue morphogenesis, angiogenesis, and tumor progression (52, 53).

**Figure 4:**
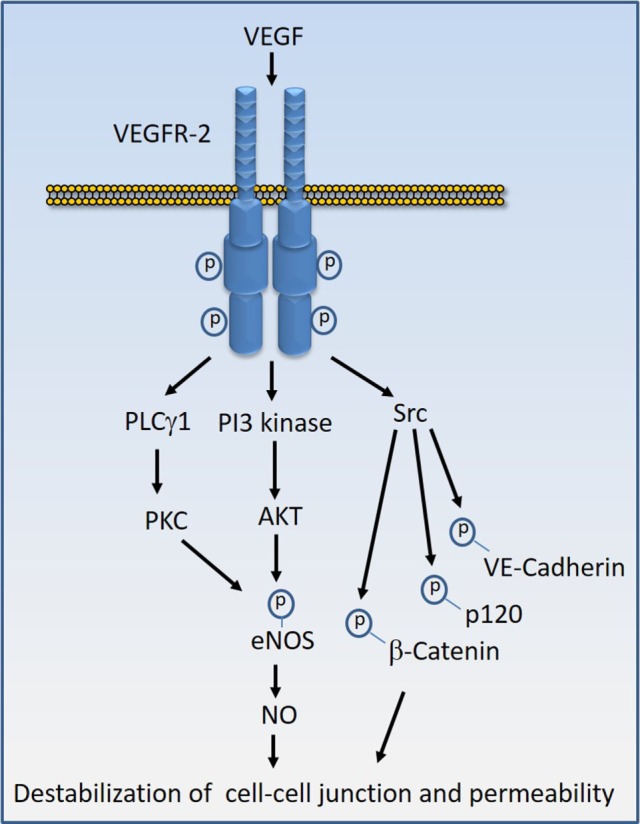
Vascular endothelial growth factor (VEGF)-induced VEGF receptor-2 (VEGFR-2) activation signal transduction that leads to destabilization of cell–cell junctions. Stimulation of VEGFR-2 by VEGF results in the kinase activation of VEGFR-2 and recruitment of diverse signaling proteins to VEGFR-2. The key VEGFR-2 signaling proteins whose activity are linked to vascular permeability include Src family kinases, phosphoinositide 3-kinase (PI3 kinase), and phospholipase Cγ1 (PLCγ1). Src kinases in turn can phosphorylate VE-cadherin and VE-cadherin-associated proteins such as β-catenin and p120 leading destabilization of VE-cadherin mediated endothelial barrier function. Activation of PI3 kinase and PLCγ1 can lead to phosphorylation of eNOS and production of nitric oxide (NO) that leads to interruption of endothelial junctions.

Various proteins and molecules could destabilize endothelial barrier function and stimulate vascular permeability. Proteins and molecules such as Ang2, chemokines, and IL-8 (interleukin-8), bradykinin, histamine, thrombin, fibrinogen, tumor necrosis factor-α (TNF-α), and endotoxins such LPS could destabilize endothelial barrier. However, VEGF, also called vascular permeability factor, is perhaps the most potent factor involved in the disruption of endothelial barrier function and induction of vascular permeability in pathological conditions (16, 54–56). In tumorigenesis, not only does VEGF induce angiogenesis but also mediates disruption of the vascular barrier, resulting in the leaky vessels leading to an increase in tumor cell extravasation and reduced drug delivery to tumor site which is associated with the development of drug resistance and inefficacy (57). Similarly, VEGF causes vascular permeability and edema in various other diseases such as diabetic retinopathy, age-related macular degeneration, and inflammation (58–61). One of VEGF’s receptor, VEGF receptor-2 (VEGFR-2), predominantly mediates VEGF-mediated destabilization of endothelial junctions (16, 62). Upon activation by VEGF, VEGFR-2 undergoes various posttranslational modifications including phosphorylation and methylation, which stimulate its activation and recruitment of signaling proteins to the receptor (63–65). Activation of VEGFR-2 by VEGF stimulates diverse signaling events that affect endothelial cell migration, proliferation, tube formation, and regulation of endothelial junctions. However, the activation of Src family kinases, phosphoinositide 3-kinase (66, 67), and phospholipase Cγ1 (PLCγ1) in particular play major roles in the induction of vascular permeability (16, 67) (Figure 4). In addition, VEGFR-2 can also stimulate permeability by directly targeting endothelial junctional proteins such as VE-cadherin and integrins (16, 62, 68, 69), providing an additional layer of complexity to VEGF-mediated destabilization of endothelial barrier function.

Vascular endothelial cadherin (VE-cadherin also called Cadherin-5 and CD144) is considered a main transmembrane component of endothelial adherens junction (70, 71). Similar to E-cadherin, VE-cadherin binds to members of the armadillo repeat family of proteins, p120-catenin, β-catenin, and plakoglobin through its cytoplasmic C-terminal (72). Inactivation of the VE-cadherin gene in both mouse and zebrafish clearly demonstrated its key role in vascular remodeling (73, 74). VE-cadherin plays an important role in controlling endothelial monolayer permeability and angiogenesis. VEGF-induced tyrosine phosphorylation of VE-cadherin at Y658 and Y731 by Src family kinases appear to play a prominent role in the destabilization of adherens junction and increased permeability of endothelial cells (75, 76). Consistent with the regulatory role of phosphorylation on VE-cadherin, other factors such as TNF-α that stimulate permeability also target VE-cadherin through tyrosine phosphorylation at Y658 and Y731 through proline-rich tyrosine kinase 2 and Rac1/Tiam1 (77). Phosphorylation of Y658 and Y731 disrupt VE-cadherin binding with VE-cadherin-associated proteins such as p120-catenin and β-catenin (78). Underscoring the role of phosphorylation in the regulation of VE-cadherin function, several protein tyrosine phosphatases are known to associate with and dephosphorylate VE-cadherin (79, 80). In view of the fundamental role of VEGF in angiogenesis and its robust action in the destabilization of endothelial barrier function in pathological conditions, VEGF system emerged as a major challenger and provocateur of the endothelial barrier function.

## IGPR-1 is a Distinct CAM

**Figure 5:**
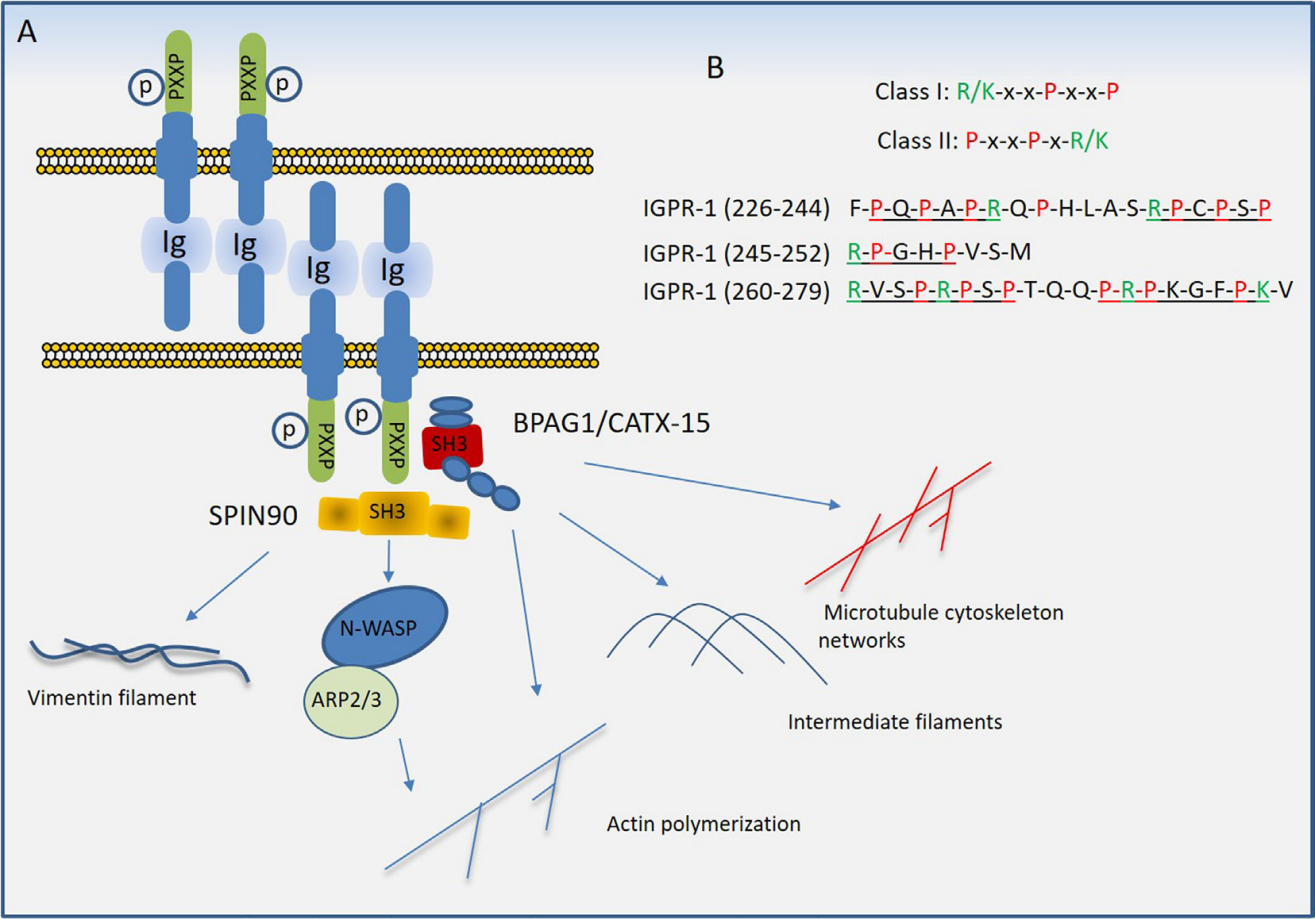
Proposed model of immunoglobulin (Ig) and proline-rich receptor-1 (IGPR-1) mediated regulation of endothelial barrier. **(A)** *Trans*-dimeric IGPR-1 undergoes serine phosphorylation at multiple sites. IGPR-1 through its proline-rich motifs recruits SH3 containing proteins, bullous pemphigoid antigen 1 (BPAG1), and SH3 protein interacting with Nck90 (SPIN90)/WISH (SH3 protein interacting with Nck). Interaction of IGPR-1 with BPAG1 and SPIN90 links IGPR-1 to actin fibril assembly, intermediate filament formation, microtubule cytoskeleton networks, and vimentin filament assembly (see text). **(B)** The conventional proline-rich motifs and the proline-rich motifs on the cytoplasmic domain of IGPR-1 are shown.

Immunoglobulin (Ig) and proline-rich receptor-1 is expressed in human endothelial and epithelial cells. Unlike the classical cadherins and tight junction proteins such as JAMs, claudins family proteins, and occludin, IGPR-1 expression is restricted to higher mammalians as it is not present in rodents such as mouse or rat (81). However, its closely related protein, transmembrane and immunoglobulin domain1 is expressed in the renal epithelial cells of human and rodents (82). IGPR-1 colocalizes with VE-cadherin in endothelial cells in cell culture and mediates endothelial cell–cell adhesion and its activity is required for angiogenesis *in vitro* and regulation of cell migration (81). Further studies revealed that it plays an important role in monolayer permeability (83). IGPR-1 is composed of three major domains: extracellular, transmembrane, and intracellular. The extracellular domain of IGPR-1 contains a single Ig domain followed by a single transmembrane domain and a proline-rich intracellular domain (81). The Ig-containing extracellular domain is required for IGPR-1 to mediate endothelial cell–cell interaction and barrier function (83). IGPR-1 is typically present as a disulfide bound *cis*-dimer, which further forms a *trans*-dimer complex in a cell density-dependent manner (83) (Figure 5A).

Proline rich sequences (PRDs) play a major role in mediating protein–protein interaction in prokaryotes and eukaryotes (84). PRDs are highly versatile and recognize different consensus motifs or canonical sequences in their protein ligands. A conspicuous feature of most binding motifs identified for PDRs is the presence of one or more proline residue that interact with the ligand, while residues that flank the core proline residue determines the selectivity (85). Although various protein domains are known to interact with proline-rich sequences (85), Src homology domain 3 (SH3) and WW domains are the most common domains that interact with PRDs (86). The PRDs of IGPR-1 interact with multiple SH3 domain-containing proteins including SPIN90/WISH (SH3 protein interacting with Nck) and bullous pemphigoid antigen 1 (BPAG1) (81). The cytoplasmic domain of IGPR-1 contains at least five PRDs (Figure 5B), which are variants of canonical class I (R/KxxPxxP) and class II (PxxPxR/K) PRD motifs (85). Furthermore, SH3 domain-containing proteins can interact with PRDs beyond the PXXP motifs, consistent with their versatility in their interaction with other proteins (85, 87).

In addition to being rich in proline residues, the cytoplasmic domain of IGPR-1 also is heavily phosphorylated at serine residues. A recent liquid chromatography–tandem mass spectrometry analysis of IGPR-1 identified seven phosphorylated serine residues on the cytoplasmic domain of IGPR-1, including Ser186, Ser220, Ser238, Ser243, Ser249, Ser262, and Ser266, five of which are located in the proline-rich region (83). Although the functional importance of these phosphorylation sites remains to be determined, phosphorylation of Ser220 is regulated by homophilic *trans*-dimerization of IGPR-1 and is required for endothelial barrier function and angiogenesis (83).

## IGPR-1 Signal Transduction in Endothelial Cells

Although significant work is required to fully understand the signal transduction events orchestrated by IGPR-1, recent studies, however, provide important new insights about signaling of IGPR-1 in endothelial cells (81, 83). Through the screening of a Src-homology3 (SH3) domain array, BPAG1 (or BP230), also called DST and SPIN90/WISH (SH3 protein interacting with Nck), also called NCKIPSD were identified as putative IGPR-1-binding proteins (81). The binding of BPAG1 and SPIN90 with IGPR-1 was further confirmed by recombinant GST-SH3 domain of BPAG1 and SPIN90 in a GST-pull down assay (81).

### Bullous Pemphigoid Antigen 1

**Figure 6:**
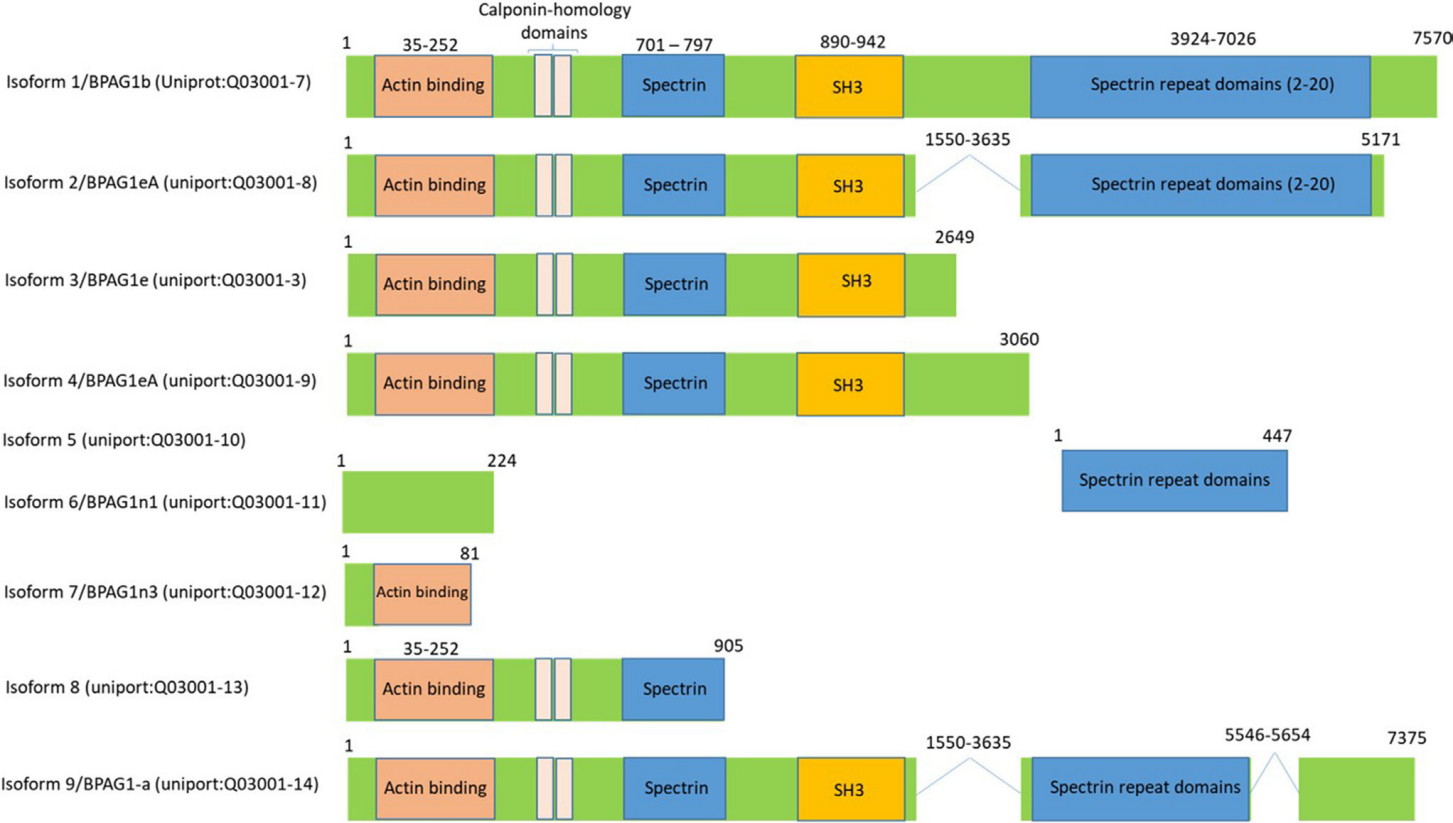
Bullous pemphigoid antigen 1 (BPAG1) is a multidomain protein with various alternatively spliced variants. BPAG1 is a large protein with 7,570 amino acids with multiple domains including N-terminus actin-binding domain, followed by plakin domain which consists of 4–8 spectrin repeats interrupted by a Src-homology3 (SH3) domain. The C-terminal of BPAG1 is composed of additional plakin repeat domains and intermediate filaments binding domain. Various alternatively spliced variants of BPAG1 are also shown.

Bullous pemphigoid antigen 1 is a member of the plakin family proteins, which include desmoplakin, plectin, envoplakin, and periplakin, is involved in cytoskeletal organization (88). BPAG1 is a cytoskeletal linker protein that crosslinks cytoskeletal filaments to membrane-associated complexes at cell junctions in epithelial cells and other cell types (89, 90). BPAG1 is a gigantic protein with 7,570 amino acids and an approximate molecular weight of 834 kDa. However, it is expressed in a various isoforms by mechanism of mRNA alternative splicing, which results in the transcription and translation that generates different isoforms of BPAG1 with varying molecular weights (91). Based on the human genome sequence information, there are 35 different transcripts of BPAG1, many of which are untranslated^1^ and based on the available human protein sequence data^2^ there are at least nine isoforms of BPAG1 (Figure 6). BPAG1 is a multidomain protein. It has a conserved N-terminal actin-binding domain, followed by plakin domain which consists of 4–8 spectrin repeats interrupted by a Src-homology3 (SH3) domain. This unique domain is conserved in all plakin family proteins. The C-terminal of BPAG1 composed of additional plakin repeat domains and intermediate filaments binding domain (88, 92–94).

Despite extensive studies on the functional role of BPAG1 in epithelial cells, expression and importance of BPAG1 in endothelial cells remains virtually unknown. However, analysis of publically available gene array datasets^3^ indicates that BPAG1 is likely expressed in mouse and human endothelial cells. Based on various recently published gene array analyzes, BPAG1 appears to widely expressed in human vascular endothelial cells derived from lung (95), macrovascular umbilical vein endothelial cells (96), umbilical cord arterial and venous endothelial cells (97), and mouse neonatal retinal endothelial cells (98), suggesting a functional role for BPAG1 in IGPR-1-mediated signal transduction in endothelial cells. Interestingly, one of the major characteristics of epidermolysis bullosa, a neurological condition that causes the skin to blister, is caused by a genetic defect in BPAG1 and it is associated with increased pathological angiogenesis with a leaky vessel (99). Nevertheless, the role of vascular component in epidermolysis bullosa remains unexamined. While the role of BPAG1 in endothelial cells and its possible role in connecting IGPR-1 cytoskeletal filaments remains unresolved, a recent study demonstrated that plectin/epiplakin 1, a closely related protein to BPAG1, is expressed in endothelial cells, which crosslinks vimentin to the actin assembly to regulate vascular integrity (100).

### SH3 Protein Interacting with Nck90

SH3 protein interacting with Nck90/WISH (SH3 protein interacting with Nck 90, also called NCKIPSD and DIP, mDia-interacting protein) is another cytoplasmic signaling protein that interacts with IGPR-1 in endothelial cells (81). SPIN90 is involved in actin polymerization *via* its interactions with Arp2/3, N-WASP, and actin (101), regulates stress fiber formation (102), Rac-mediated membrane ruffling (103), and binds to Palladin, a cytoskeletal protein that is required for organization of normal actin cytoskeleton, which is important for cell morphology, motility, and cell adhesion (104). SPIN90 is highly expressed in endothelial cells and the siRNA-mediated downregulation of SPIN90 inhibited capillary tube formation, suggesting an important role for SPIN90 in IGPR-1-mediated signaling events in endothelial cells and angiogenesis (81).

Although a significant work remains, the emerging picture of IGPR-1-mediated signal transduction in endothelial cells indicates that IGPR-1 is cross-linked to actin fibril assembly and other cytoskeletal filaments that contributes to endothelial cell adhesion, integrity, and barrier function partly through interaction with SPIN90 and BPAG1. However, deciphering the molecular mechanisms of IGPR-1 in various cell culture systems and animal models other than mouse (IGPR-1 is not expressed mouse or rat) is an important area for future research, which may lead to the discovery of new therapeutic targets for various human diseases associated with endothelial dysfunction.

## Conclusion

Altered endothelial barrier function is a hallmark of many human disorders. Understanding the molecular mechanisms of vascular permeability could lead to new therapeutic strategies to prevent vascular leakage and improve drug delivery. Moreover, controlling integrity and function of endothelial cells in organ transplantation could reduce complications associated with transplantation medicine.

## Author Contributions

The author confirms being the sole contributor of this work and approved it for publication.

## Conflict of Interest Statement

The author declares that the research was conducted in the absence of any commercial or financial relationships that could be construed as a potential conflict of interest.

## Footnotes

^1^http://www.ensembl.org.

^2^http://www.uniprot.org/.

^3^https://www.ncbi.nlm.nih.gov/geo/.
